# Effects of maternal geohelminth infections on allergy in early childhood

**DOI:** 10.1016/j.jaci.2015.07.044

**Published:** 2016-03

**Authors:** Philip J. Cooper, Martha E. Chico, Leila D. Amorim, Carlos Sandoval, Maritza Vaca, Agostino Strina, Ana Clara Campos, Laura C. Rodrigues, Mauricio L. Barreto, David P. Strachan

**Affiliations:** a Laboratorio de Investigaciones FEPIS, Avenida Via Guayllabamba, Quininde, Ecuador; b Centro de Investigacion en Enfermedades Infecciosas, Pontificia Universidad Catolica del Ecuador, Quito, Ecuador; c Institute of Infection and Immunity, St George's University of London, London, United Kingdom; d Institute of Public Health Sciences, St George's University of London, London, United Kingdom; e Instituto de Saude Coletiva, Universidade Federal da Bahia, Rua Basilio de Gama, Salvador, Brazil; f Faculty of Epidemiology and Population Health, London School of Hygiene and Tropical Medicine, London, United Kingdom

**Keywords:** Geohelminths, maternal infections, atopy, wheeze, eczema, early childhood, epg, Eggs per gram, HDM, House dust mite, OR, Odds ratio, SPT, Skin prick test

## Abstract

**Background:**

Maternal geohelminth infections during pregnancy may protect against allergy development in childhood.

**Objective:**

We sought to investigate the effect of maternal geohelminths on the development of eczema, wheeze, and atopy during the first 3 years of life.

**Methods:**

A cohort of 2404 neonates was followed to 3 years of age in a rural district in coastal Ecuador. Data on wheeze and eczema were collected by means of questionnaire and physical examination at 13, 24, and 36 months of age. Atopy was measured based on skin prick test (SPT) reactivity to 9 allergens at 36 months. Maternal stool samples were examined for geohelminths by microscopy. Data on potential confounders was collected after birth by questionnaire.

**Results:**

Geohelminths were observed in 45.9% of mothers. Eczema and wheeze were reported for 17.7% and 25.9%, respectively, of 2069 (86.1%) children with complete follow-up to 3 years, and allergen SPT reactivity to any allergen was present in 17.2% and to house dust mite in 8.7%. Maternal geohelminth infections were not significantly associated with eczema (adjusted odds ratio [OR], 1.26; 95% CI, 0.98-1.61), wheeze (adjusted OR, 1.02; 95% CI, 0.82-1.27), and SPT reactivity to any allergen (adjusted OR, 0.79; 95% CI, 0.61-1.01). In subgroup analyses maternal geohelminths were associated with a significantly reduced risk of SPT reactivity to mite and other perennial allergens, and maternal ascariasis was associated with an increased risk of eczema and reduced risk of SPT reactivity to all allergens.

**Conclusion:**

Our data do not support a protective effect of maternal infections with geohelminth parasites during pregnancy against the development of eczema and wheeze in early childhood, although there was evidence in subgroup analyses for a reduction in SPT reactivity to house dust mites and perennial allergens.

Allergic diseases, such as asthma and eczema, have emerged as major health problems in many developing countries, particularly in urban Latin America.^1^ The causes of such temporal increases are likely to be explained by environmental changes.^1^, ^2^

The hygiene hypothesis has explained the increasing prevalence of allergic diseases in the context of decreasing exposures to common infectious diseases of childhood^3^ and a decreased diversity of microbial exposures in the environment.^4^ Among potentially protective infections, there has been considerable interest in the role of geohelminth parasites (eg, *Ascaris lumbricoides*, *Trichuris trichiura*, and hookworm), which are extremely common chronic infections of childhood in developing countries, where an estimated 2 billion humans are infected. The relationship between geohelminth infections and allergy is complex.^5^ Although infections are generally associated with a reduced prevalence of allergen skin prick test (SPT) reactivity,^6^ their effects on allergic diseases are less clear.^7^ Treatment studies of geohelminths in schoolchildren have not shown effects on the prevalence of allergic diseases^8^, ^9^, ^10^ or consistent increases in allergen SPT reactivity prevalence^8^, ^9^, ^10^, ^11^ after anthelmintic treatment. It has been suggested that *in utero* and early-life exposures might be important for mediating protection.^12^ Other environmental exposures that have been associated with protection against allergy, such as farming, have stronger effects when exposure occurs *in utero* and in early childhood.^13^, ^14^, ^15^

We recruited a birth cohort in an area of Ecuador with a high maternal prevalence of geohelminth infections to test the hypothesis that *in utero* exposures to geohelminth parasites through having an infected mother protect against the development of eczema, wheeze, and atopy during the first 3 years of life.

## Methods

### Study design and setting

A prospective study from birth was done in the rural District of Quininde, Esmeraldas Province, Ecuador, as described in detail elsewhere.^16^ The district serves a population of approximately 150,000 with an economy based on agricultural activities, primarily African palm oil, and limited access to basic services.

### Study participants

Neonates were recruited in the public hospital, Hospital “Padre Alberto Buffini,” which serves the district, between November 2005 and December 2009. Eligibility criteria were as follows: (1) healthy baby aged less than 14 days; (2) collection of stool sample from the mother; (3) mother aged 17 years or more; (4) family resident in the District of Quininde for at least 2 years with plans to stay for at least 3 more years; and (5) accessible household.

### Questionnaires

Children were followed up to 3 years of age. Data on demographic and socioeconomic factors, household characteristics, maternal obstetric history, family history of chronic illnesses, and relevant environmental exposures within and outside the household were collected using an interview-led questionnaire from the child's mother after the child's birth. Questionnaires were done annually at 13, 24, and 36 months to collect data on the development of eczema and wheezing and other relevant information.

### Measurement of maternal infections with geohelminth infections

Stool samples to measure geohelminth infections were collected from mothers during the third trimester or immediately after the child's birth and in children at 13 and 24 months of age. Samples were examined using a combination of methods, including saline mounts, the Kato-Katz method, formol-ether concentration, and carbon coproculture methods.^17^ A positive sample was defined by the presence of at least 1 egg or larva from any of the 4 detection methods. *A lumbricoides* and *T trichiura* infection intensities were expressed as eggs per gram (epg) of feces using results from the Kato-Katz method.^18^

### Measurement of eczema and wheeze

Data were collected using periodic questionnaires and examinations for visible signs of flexural dermatitis with a standardized photographic protocol.^18^ Eczema was defined using the United Kingdom refinement of the Hanifin and Rajka diagnostic criteria.^18^ Children were considered to have eczema if the mother reported the child to have had an itchy skin condition during the previous 12 months plus at least 3 of the following: (1) involvement of skin creases and cheeks; (2) history of allergic disease in siblings or parents; (3) history of generally dry skin; and (4) visible flexural dermatitis, including that affecting the cheeks, forehead and outer limbs. Recent wheeze was defined as any episode of reported wheeze during the previous 12 months.

### Allergen SPT reactivity

Allergic sensitization was measured at 36 months using SPTs with 9 allergen extracts (Greer Laboratories, Lenoir, NC): house dust mites (HDMs; *Dermatophagoides pteronyssinus*/*Dermatophagoides farinae* mix), American cockroach *(Periplaneta americana)*, cat, dog, grass pollen (9 southern grass mix), fungi (new stock fungi mix), egg, milk, and peanut, with positive histamine and negative saline controls. A positive reaction was defined as a mean wheal diameter of at least 2 mm greater than that elicited by the saline control 15 minutes after pricking the allergen onto the volar side of the forearm with ALK-Abelló lancets (ALK-Abelló, Hungerford, United Kingdom). A positive SPT response was defined as a positive reaction to any of the allergens tested. All testing was done by trained physicians (M.E.C. and M.V.).

### Statistical analysis

We estimated that with 1840 children followed up at 3 years of age and approximately 50% of mothers infected with geohelminths, we would have greater than 95% power to detect a difference of at least 5% between groups for outcomes with 10% or greater prevalence, considering a *P* value of less than .05. The primary analysis was the association between maternal geohelminths and eczema, wheeze, and atopy. Secondary analyses addressed associations between maternal geohelminth species and study outcomes, including mite atopy. Bivariate logistic regression analyses were done to estimate associations between maternal geohelminth infections or potential confounders and outcomes. Multivariate logistic regression was used to estimate associations between maternal geohelminth infections and outcomes. Potential confounders considered in the analyses are shown in Table I. A socioeconomic status index was created by using principal components analysis of 7 socioeconomic variables, as previously described.^19^ Child geohelminth infections were defined as any infection during the first 2 years of life. Potential confounders in any of the bivariate analyses with a *P* value of less than .20 were kept in the final models using the same set of confounders to adjust all models. All statistical analyses were done with Stata 11 software (StataCorp, College Station, Tex).

### Ethical considerations

The protocol was approved by the ethics committees of the Hospital Pedro Vicente Maldonado, Universidad San Francisco de Quito, and Pontificia Universidad Catolica del Ecuador. The study is registered as an observational study (ISRCTN41239086). Informed written consent was obtained from the child's mother. Anthelmintic treatment (single dose of 400 mg of albendazole) was provided to mothers with geohelminth infections after delivery. Children with positive stool results for geohelminths were treated with a single dose of 400 mg of albendazole if aged 2 years or greater and with pyrantel pamoate (11 mg/kg) if aged less than 2 years, according to Ecuadorian Ministry of Public Health recommendations.^20^, ^21^

## Results

### Cohort participants

**Fig 1:**
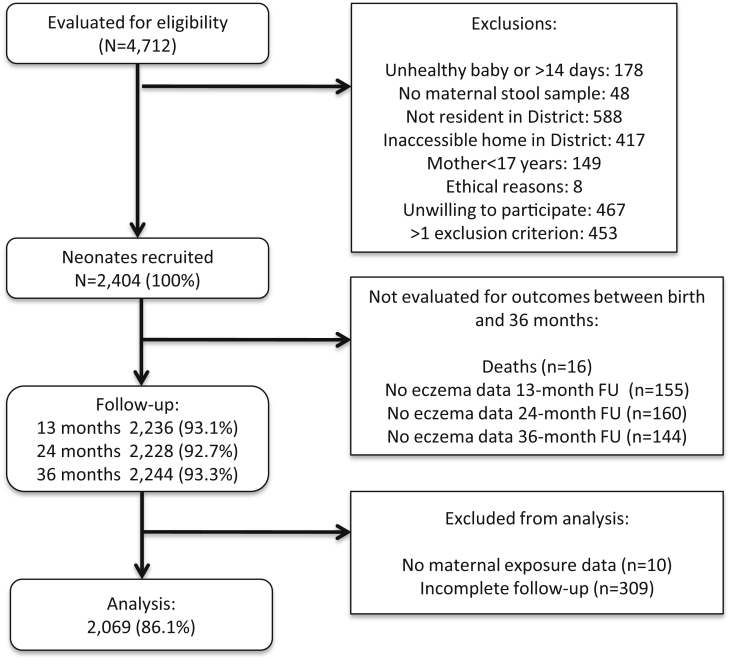
Participant flow through follow-up to 3 years of age and those included in the analysis.

Follow-up information for the 2404 neonates recruited is provided in Fig 1. Follow-up was greater than 90% at each of the annual follow-ups during the first 3 years of life in the cohort, and complete data for all 3 observation times (ie, 13, 24, and 36 months) were available for 2069 (86.1%) children. Maternal infections with malaria, HIV, and other helminths were of low prevalence (<0.5%) in the study population.

### Frequencies of exposures and outcomes

Geohelminth infections were detected in 45.9% of mothers as follows: *A lumbricoides* (27.5%), *T trichiura* (28.9%), hookworm (5.6%), and *Strongyloides stercoralis* (3.9%). The only other helminth parasite observed was the cestode *Hymenolepis nana* (0.5%). The proportions of mothers by infection intensity category were as follows: *A lumbricoides* light (<5000 epg) at 17.1% and moderate/heavy (>5000 epg) at 5.1% and *T trichiura* light (<1000 epg) at 22.0% and moderate/heavy (>1000 epg) at 4.2%. The prevalence of eczema at 13, 24, and 36 months was 12.6%, 4.7%, and 3.4%, respectively, with 17.7% of children having eczema at least once. Only 2.5% had episodes of eczema documented for 2 or more visits. The prevalence of reported wheeze at 13, 24, and 36 months was 16.1%, 8.0%, and 8.5%, respectively, and 25.9% had at least 1 reported episode. Allergen SPT reactivity was present in 17.2% of children at 3 years of age: *D pteronyssinus*/*D farina*e (8.7%), cockroach (2.9%), mixed fungi (2.4%), dog (2.3%), cat (1.2%), mixed grasses (2.5%), peanut (1.2%), milk (1.2%), and egg (1.9%).

### Determinants of wheeze, eczema, and allergen SPT reactivity between birth and 3 years of age

The distributions of demographic and potential confounding variables are shown in Table I. Most mothers were of Mestizo ethnicity (73.4%), had at least completed primary education (73.4%), and lived in a neighborhood (70.5%) of one of the towns (denoted urban) in the district. Almost a third (28.9%) of children acquired at least 1 geohelminth infection during the first 2 years of life: *A lumbricoides* in 22.6% and *T trichiura* in 12.7%. Prevalence of any geohelminth infections at 13 and 24 months were 12.3% and 21.8%, respectively. Table I shows results of bivariate analyses between exposures or potential confounders and study outcomes. Results of multivariate analyses are shown in Table II. None of the outcomes in children up to or at 3 years of age were statistically significantly associated with maternal geohelminth infections after adjustment for potential confounders. With respect to other risk factors, maternal allergy (odds ratio [OR], 2.29), household pets (OR, 1.32), and infantile pneumonia (OR, 2.05) were associated with eczema. Having an Afro-Ecuadorian mother (vs others; OR, 1.30) and infantile pneumonia (OR, 5.64) were associated with wheeze. SPT response positivity to any allergen was not significantly associated with any of the variables analyzed. Infection with any geohelminth parasite during the first 2 years of life was not associated with any of the outcomes measured at 3 years of age.

For the analysis presented here, we considered only complete data for all observation times to reduce classification bias for those with incomplete data. We compared risk factors between the subjects included in the analysis with those with incomplete information and found no substantial differences between the 2 groups. Thus we can assume that missing data were random and should not have led to bias.

### Effects of maternal geohelminths on SPT reactivity to HDMs and perennial allergens

Because HDMs are the dominant allergens in our study population, we analyzed separately the associations of maternal geohelminths and potential confounders with SPT reactivity to HDM and all perennial allergens tested. Results of crude and adjusted analyses are provided in Table E1 in this article's Online Repository at www.jacionline.org. In adjusted analyses maternal geohelminth infections were significantly inversely associated with SPT reactivity to HDM (OR, 0.61) and all perennial allergens (OR, 0.69). SPT reactivity to HDM was independently associated with maternal contact with large farm animals during pregnancy (OR, 0.72).

### Effects of individual geohelminth parasites on study outcomes

Table E2 in this article's Online Repository at www.jacionline.org shows the results of crude and Table III shows the results of adjusted analyses of the associations between individual geohelminth species detected in the mother and outcomes. In adjusted analyses *A lumbricoides* infection was significantly associated with an increased prevalence of early childhood eczema (OR, 1.36) but a lower prevalence of SPT reactivity to any allergen (OR, 0.70) and HDM (OR, 0.48). There was a borderline significantly increased risk of wheeze associated with *A lumbricoides* infection (OR, 1.27). *T trichiura* and hookworm infections were not associated with eczema, wheeze, or SPT response positivity to any allergen or to HDM. There was no evidence of stronger effects at higher parasite burdens of maternal *A lumbricoides*.

## Discussion

In this prospective study done from birth to 3 years of age in a rural district of Esmeraldas Province in a tropical region of Ecuador, we have examined the hypothesis that maternal geohelminth infections during pregnancy protect against the development of atopy (measured using SPTs), eczema, and wheeze in early childhood. Overall, our data indicate that maternal geohelminths do not protect against eczema and wheeze in early childhood. In subgroup analyses there was evidence that maternal geohelminths protected against SPT reactivity to mite and other perennial allergens and that maternal ascariasis increased the risk of eczema but reduced the prevalence of allergen SPT reactivity.

Helminth parasites are potent modulators of the host immune response.^22^ Their modulatory effects are directed largely against allergic inflammatory mechanisms that serve to kill the parasites and prevent establishment of chronic infections or control parasite numbers once chronic infections have become established.^22^, ^23^ It is widely believed that the immune modulation associated with chronic helminth infections might also protect against allergic inflammation and allergic diseases.^22^, ^23^ Numerous studies have shown geohelminths to be inversely associated with SPT response positivity in schoolchildren,^6^ although the effects of geohelminths on allergic diseases are less consistent.^7^ The treatment of geohelminths does not appear to affect the prevalence of allergic diseases^8^, ^9^, ^10^ but has been shown to increase allergen SPT reactivity in some^9^, ^11^ but not other^8^, ^10^ studies. One explanation for the inconsistent findings of randomized treatment studies on allergen SPT reactivity is the presence of different geohelminth parasites in different populations that might have distinct effects on allergy. Another is that the protective effects of geohelminths against allergy can be programmed in early life through *in utero* exposures to parasite antigens from infected mothers, through the effects of infections acquired in early childhood, or both. Certainly, allergic diseases are considered to start in early life, and the effects of protective environmental exposures, such as farming, might be strongest when they occur *in utero*, in the first year of life, or both.^15^

Our findings do not support a protective effect of maternal geohelminth infections against the development of eczema and wheezing illness in early childhood. It is possible that the atopic phenotype is not fully developed by 3 years of age, and future evaluations of the cohort in later childhood might be required to more fully understand the effects of maternal geohelminths on allergic disease. To our knowledge, the only other published study of the effects of maternal geohelminths on allergy was a study in Uganda that showed that treatment of mothers in the second or third trimester of pregnancy with a single dose of albendazole was associated with an increased risk of doctor-diagnosed eczema during the first year of life.^24^, ^25^ Surprisingly, the effect of treatment with albendazole on eczema risk appeared to be greater in infants of mothers without than those with hookworm infection.^25^ Possible explanations for differences in findings between the Ugandan study and our own include (1) effects of maternal albendazole on organisms other than hookworm or a direct effect on the developing immune system^25^ and (2) helminth-specific effects: hookworm, which infected only 5.6% of mothers in our study, and was the predominant geohelminth in the Ugandan study (44% of mothers^25^), appears to protect against the risk of asthma in older children^7^ and might also reduce the risk of eczema in infancy^24^ and might be the only common geohelminth with protective effects against allergic diseases.

We have shown previously in a cross-sectional analysis of schoolchildren in the same Ecuadorian province that childhood infections with geohelminths did not protect against symptoms of eczema, rhinitis, and wheeze but were inversely associated with allergen SPT reactivity.^26^ Symptoms of wheeze and rhinitis were associated with poor hygiene exposures,^26^ whereas a study in urban Brazil showed that childhood asthma was associated with living in “dirty” households.^27^ Symptoms of so-called allergic diseases in these populations were weakly associated with allergen SPT reactivity.^26^, ^27^ Such attenuation might be explained by the immune-modulatory effects of early-life exposures to geohelminths and a microbially diverse environment.

In analyses of subgroups within the cohort, we observed a protective effect for maternal geohelminths against the development of SPT reactivity to HDM and other perennial allergens and for maternal ascariasis against SPT reactivity to any allergen at 3 years of age. These effects were not related to infection intensities (for maternal ascariasis) and were not attributable to the acquisition of geohelminth infections by the children during the first 2 years of life. Our data extend the findings of previous studies done in children^5^, ^28^ to raise the possibility that the postulated protective effect of geohelminths against allergen SPT reactivity might originate much earlier in life than previously shown.

We also observed a protective effect of exposure to farm animals against SPT reactivity to HDM. Farm animal exposures were measured during the first home visit between birth and 2 weeks of age and likely represent exposure during pregnancy and early childhood, assuming no later changes in the circumstances of the child's household. Previous studies have shown that living on a farm during pregnancy protects against atopy and allergic diseases,^13^, ^14^ an effect attributed to the wide variety of microbial exposures of farm residence,^29^ leading perhaps to the induction of robust immune regulation.^4^

The strengths of the study include its prospective design and the collection of a large number of sociodemographic and lifestyle variables allowing us to control for potential confounders: the fact that ORs were affected little by adjustment indicates minimal residual confounding. Potential biases were reduced by using objective measures of geohelminth infection, performing all evaluations blind to the child's exposure status, and high rates of retention in the cohort to 3 years of age. Repeated collection of data over the 3 years by experienced and trained clinicians provided more precise estimates of the prevalence of wheeze and eczema. Some degree of misclassification of eczema with other skin conditions, such as scabies, cannot be excluded, although the eczema definition used here^18^ might be more specific than other definitions, such as that used by the International Study of Asthma and Allergies in Childhood.^30^ Previous studies that have addressed the effects of geohelminths during early childhood on the development of eczema or wheeze in early childhood either were unable to measure associations^31^ or had limited power^24^ because of low prevalence. Maternal geohelminth infections were measured in the third trimester or in a stool sample collected around the time of birth, the latter being a reasonable proxy for the former because these infections are chronic and can survive in the human intestinal tract for several years.

In summary, we have examined the effects of exposures to geohelminths in pregnancy and early childhood on the development of eczema, wheeze, and allergen SPT reactivity. Our data do not support the hypothesis that early-life exposures to geohelminths protect against the development of eczema and wheeze. Similarly, there was no significant overall protective effect of maternal geohelminths against allergen SPT reactivity, although in subgroup analyses there was evidence for protection against SPT reactivity to HDM and perennial allergens and that maternal ascariasis protected against allergen SPT reactivity, the clinical significance of which remains to be defined and will require replication in future studies.

**Key messages**

• *In utero* exposures to geohelminths are considered to protect against allergy and contribute to the low prevalence of allergic diseases in the rural tropics.
• Data from a birth cohort in Ecuador did not show a significant protective effect of maternal geohelminths against the development of eczema, wheeze, and atopy during the first 3 years of life.
• Subgroup analyses indicated a possible protective effect of maternal geohelminths against atopy to perennial allergens, particularly mite.

## Figures and Tables

**Table E1:** Crude and adjusted analyses for associations between maternal geohelminth infections and potential confounders with SPT reactivity to HDMs and perennial allergens (any of HDM, cockroach, cat, and dog)

Variable	Overall	SPT reactivity to HDM					SPT reactivity to perennial allergens				
No. (%)	Prevalence (%)	Crude OR (95% CI)	*P* value	Adjusted OR (95% CI)	*P* value	Prevalence (%)	Crude OR (95% CI)	*P* value	Adjusted OR (95% CI)	*P* value
Any maternal geohelminth											
No	1119 (54.1)	10.2	1		1		13.9	1		1	
Yes	950 (45.9)	6.9	**0.66 (0.48-0.91)**	**.010**	**0.61 (0.43-0.86)**	**.005**	10.5	**0.72 (0.55-0.95)**	**.019**	**0.69 (0.51-0.92)**	**.012**
Maternal age (y)											
≤20	535 (25.9)	8.0	1				12.1	1			
21-29	1002 (48.4)	8.9	1.13 (0.77-1.67)	.517			12.5	1.03 (0.75-1.43)	.837		
≥30	532 (25.7)	8.9	1.14 (0.73-1.75)	.567			12.2	1.00 (0.69-1.45)	.982		
Maternal ethnicity											
Non–Afro-Ecuadorian	1524 (73.7)	8.5	1		1		12.1	1		1	
Afro-Ecuadorian	545 (26.3)	9.3	1.10 (0.78-1.55)	.578	1.11 (0.77-1.59)	.586	12.8	1.07 (0.79-1.43)	.700	1.07 (0.79-1.46)	.659
Maternal educational level											
Illiterate	317 (15.3)	8.4	1		1		11.7	1		1	
Complete primary	1203 (58.1)	8.7	1.04 (0.66-1.63)	.860	0.82 (0.50-1.34)	.419	12.5	1.08 (0.74-1.60)	.682	0.94 (0.62-1.43)	.768
Complete Secondary	549 (26.6)	8.7	1.04 (0.63-1.72)	.879	0.58 (0.32-1.05)	.070	12.2	1.06 (0.69-1.63)	.798	0.73 (0.43-1.21)	.221
Area of residence											
Urban	1458 (70.5)	9.5	1		1		13.1	1		1	
Rural	611 (29.5)	6.8	0.70 (0.49-1.01)	.055	0.83 (0.55-1.25)	.363	10.5	0.78 (0.58-1.05)	.105	0.85 (0.60-1.20)	.347
Sex											
Male	1052 (50.9)	9.0	1		1		12.9	1		1	
Female	1017 (49.1)	8.4	0.93 (0.68-1.26)	.629	0.92 (0.67-1.26)	.618	11.7	0.89 (0.68-1.16)	.383	0.88 (0.67-1.15)	.346
Socioeconomic status∗											
1	678 (32.7)		1		1		11.1	1		1	
2	679 (32.8)	6.7	1.36 (0.91-2.04)	.133	1.32 (0.88-2.02)	.181	12.2	1.12 (0.80-1.56)	.508	1.11 (0.78-1.57)	.653
3	712 (34.4)	9.0	**1.58 (1.07-2.33)**	**.020**	1.45 (0.93-2.26)	.097	13.6	1.26 (0.91-1.74)	.165	1.18 (0.82-1.71)	.376
Birth order											
First	517 (25.0)	10.4	1		1		13.6	1		1	
Second-fourth	1148 (55.5)	8.6	0.80 (0.57-1.15)	.231	0.77 (0.54-1.12)	.174	12.4	0.90 (0.66-1.23)	.504	0.85 (0.62-1.17)	.321
≥Fifth	404 (19.5)	6.8	0.63 (0.39-1.02)	.058	0.61 (0.35-1.04)	.070	10.6	0.75 (0.50-1.13)	.172	0.70 (0.44-1.10)	.125
Maternal allergy											
No	1959 (95.3)	8.6	1		1		12.2	1		1	
Yes	97 (4.7)	10.4	1.24 (0.63-2.42)	.538	1.33 (0.67-2.64)	.422	15.6	1.34 (0.76-2.36)	.318	1.40 (0.79-2.50)	.250
Household overcrowding†											
≤3	1174 (56.7)	8.9	1		1		12.1	1		1	
>3	895 (43.3)	8.4	0.93 (0.68-1.27)	.644	1.06 (0.81-1.38)	.690	12.6	1.06 (0.81-1.38)	.690	1.21 (0.90-1.63)	.208
Pets inside house											
No	1544 (74.6)	8.2	1		1		11.9	1		1	
Yes	525 (25.4)	10.0	1.24 (0.88-1.74)	.882	1.15 (0.86-1.55)	.355	13.5	1.15 (0.86-1.55)	.355	1.19 (0.88-1.61)	.260
Large farm animals‡											
No	1405 (67.9)	9.9	1		1		13.5	1		1	
Yes	664 (32.1)	6.5	**0.63 (0.45-0.89)**	**.009**	**0.72 (0.54-0.96)**	**.025**	10.1	**0.72 (0.54-0.96)**	**.025**	0.76 (0.56-1.05)	.095
Pneumonia to 13 mo											
No	1971 (95.5)	8.9	1		1		12.5	1		1	
Yes	94 (4.4)	4.3	0.45 (0.16-1.25)	.127	0.65 (0.31-1.36)	.252	8.5	0.65 (0.31-1.36)	.252	0.57 (0.26-1.26)	.166
Child geohelminths§											
No	1452 (71.1)	9.1	1		1		12.9	1		1	
Yes	590 (28.9)	7.9	0.85 (0.60-1.21)	.369	0.86 (0.64-1.59)	.320	11.3	0.86 (0.64-1.59)	.320	0.92 (0.66-1.26)	.587

ORs and 95% CIs were estimated using logistic regression. *P* values of less than .05 are shown in boldface. Adjusted ORs are controlled for maternal ethnicity and educational level, area of residence, sex, socioeconomic status, birth order, maternal allergy, household overcrowding, pets inside the house, contact with large farm animals, pneumonia to 13 months, and geohelminths in the child.

∗ Socioeconomic status *(SES)* represents tertiles of *z* scores obtained by using a factor analysis, with 1 representing the lowest and 3 representing the highest SES.

† Household overcrowding is defined as the number of persons living in the household per sleeping room.

‡ Any of cows, pigs, mules, donkeys, and horses.

§ Geohelminth infections in the child: presence of any geohelminth infection detected during the first 2 years of life.

**Table E2:** Crude associations between maternal geohelminth infections and study outcomes

Geohelminth variable	Eczema to 3 y		Wheeze to 3 y		Allergen SPT reactivity at 3 y		SPT reactivity to HDM at 3 y	
OR (95% CI)	*P* value	OR (95% CI)	*P* value	OR (95% CI)	*P* value	OR (95% CI)	*P* value
Any maternal geohelminth presence								
No (54.1%)	1		1		1		1	
Yes (45.9%)	**1.31 (1.05-1.65)**	**.018**	1.17 (0.96-1.43)	.110	0.84 (0.66-1.05)	.131	**0.66 (0.48-0.91)**	**.010**
Maternal *A lumbricoides* presence								
No (72.5%)	1		1		1		1	
Yes (27.5)	**1.44 (1.13-1.84)**	**.003**	**1.38 (1.11-1.72)**	**.003**	**0.74 (0.57-0.97)**	**.031**	**0.51 (0.34-0.77)**	**.001**
Intensity								
Uninfected (77.8%)	1		1		1		1	
Light (17.1%)	1.28 (0.92-1.78)	.150	**1.33 (1.03-1.72)**	**.027**	**0.68 (0.49-0.96)**	**.030**	**0.51 (0.31-0.84)**	**.008**
Moderate/heavy (5.1%)	1.45 (0.85-2.50)	.176	**1.63 (1.07-2.48)**	**.022**	0.88 (0.52-1.51)	.647	0.46 (0.18-1.15)	.095
Maternal *T trichiura* presence								
No (71.1%)	1		1		1		1	
Yes (28.9%)	1.21 (0.92-1.60)	.181	1.16 (0.93-1.43)	.181	0.85 (0.66-1.10)	.222	**0.65 (0.45-0.94)**	**.022**
Intensity								
Uninfected (73.8%)	1		1		1		1	
Light (22.0%)	1.20 (0.88-1.64)	.243	1.20 (0.88-1.64)	.854	0.83 (0.62-1.11)	.209	0.66 (0.44-1.00)	.053
Moderate/heavy (4.2%)	1.75 (0.99-3.58)	.053	1.75 (0.99-3.09)	.576	0.67 (0.36-1.31)	.254	**0.22 (0.05-0.92)**	**.037**
Maternal hookworm presence								
No (94.4%)	1		1		1		1	
Yes (5.6%)	1.51 (0.92-2.49)	.106	0.87 (0.56-1.36)	.541	0.79 (0.46-1.35)	.384	0.79 (0.38-1.65)	.535

ORs and 95% CIs were estimated using logistic regression. *P* values of less than .05 are shown in boldface. Uninfected data for prevalence are derived from the results of all parasitologic methods, whereas those for infection intensity form Kato-Katz results.

**Table I:** Frequencies of maternal geohelminth infections and potential confounders and associations with study outcomes

Variable	Overall	Eczema to 3 y			Wheeze to 3 y			Allergen SPT reactivity at 3 y		
No. (%)	Percent	OR (95% CI)	*P* value	Percent	OR (95% CI)	*P* value	Percent	OR (95% CI)	*P* value
Any maternal geohelminth										
No	1119 (54.1)	15.9	1		24.5	1		18.3	1	
Yes	950 (45.9)	19.9	**1.31 (1.05-1.65)**	**.018**	27.6	1.17 (0.96-1.43)	.110	15.8	0.84 (0.66-1.05)	.131
Maternal age (y)										
≤20	535 (25.9)	18.5	1		28.4	1		16.9	1	
21-29	1002 (48.4)	17.8	0.95 (0.72-1.25)	.719	25.8	0.88 (0.66-1.15)	.346	17.3	1.03 (0.78-1.36)	.843
≥30	532 (25.7)	16.9	0.90 (0.65-1.23)	.497	24.8	0.83 (0.61-1.13)	.243	17.3	1.03 (0.75-1.42)	.848
Maternal ethnicity										
Non–Afro-Ecuadorian	1524 (73.7)	16.6	1		24.3	1		16.8	1	
Afro-Ecuadorian	545 (26.3)	20.9	**1.33 (1.40-1.70)**	**.024**	30.3	**1.40 (1.09-1.68)**	**.007**	18.2	1.10 (0.85-1.42)	.480
Maternal educational level										
Illiterate	317 (15.3)	20.2	1		28.4	1		19.4	1	
Complete primary	1203 (58.1)	16.9	0.80 (0.59-1.10)	.168	25.8	0.88 (0.66-1.15)	.346	16.9	0.84 (0.61-1.16)	.294
Complete secondary	549 (26.6)	18.2	0.88 (0.62-1.25)	.475	24.8	0.83 (0.61-1.13)	.243	16.5	0.82 (0.57-1.18)	.285
Area of residence										
Urban	1458 (70.5)	17.6	1		27.0	1		17.5	1	
Rural	611 (29.5)	18.2	1.04 (0.82-1.33)	.741	23.2	0.82 (0.66-1.02)	.073	16.5	0.93 (0.72-1.20)	.592
Sex										
Male	1052 (50.9)	18.3	1		28.3			17.9	1	
Female	1017 (49.1)	17.2	0.93 (0.74-1.17)	.535	23.4	**0.77 (0.63-0.94)**	**.011**	16.5	0.91 (0.72-1.14)	.400
Socioeconomic status∗										
1	678 (32.7)	19.8	1		27.6	1		16.8	1	
2	679 (32.8)	16.6	0.81 (0.61-1.07)	.137	23.0	0.78 (0.61-1.00)	.051	17.2	1.03 (0.77-1.37)	.846
3	712 (34.4)	16.9	0.82 (0.63-1.08)	.161	27.1	0.98 (0.77-1.24)	.843	17.6	1.06 (0.80-1.40)	.702
Birth order										
First	517 (25.0)	17.0	1		22.2	1		18.9	1	
Second-fourth	1148 (55.5)	18.4	1.10 (0.83-1.44)	.504	26.7	1.27 (0.99-1.62)	.056	17.0	0.89 (0.68-1.17)	.401
≥Fifth	404 (19.5)	16.8	0.99 (0.70-1.40)	.939	28.5	**1.39 (1.03-1.88)**	**.031**	15.8	0.82 (0.58-1.16)	.264
Maternal allergy										
No	1959 (95.3)	16.8	1		25.6	1		17.0	1	
Yes	97 (4.7)	33.0	**2.44 (1.57-3.79)**	**<.001**	32.0	1.36 (0.88-2.11)	.166	20.8	1.29 (0.77-2.13)	.332
Household overcrowding†										
≤3	1174 (56.7)	16.1	1		24.0	1		17.0	1	
>3	895 (43.3)	19.9	**1.29 (1.03-1.62)**	**.026**	28.4	**1.25 (1.03-1.53)**	**.025**	17.4	1.03 (0.81-1.30)	.820
Pets inside house										
No	1544 (74.6)	16.8	1		25.4	1		17.3	1	
Yes	525 (25.4)	20.6	1.28 (1.00-1.65)	.050	27.4	1.11 (0.89-1.39)	.357	16.9	0.98 (0.75-1.27)	.865
Large farm animals‡										
No	1405 (67.9)	17.7	1		26.7	1		18.3	1	
Yes	664 (32.1)	17.9	1.02 (0.80-1.30)	.881	24.3	0.88 (0.71-1.09)	.236	14.8	**0.77 (0.60-1.00)**	**.048**
Pneumonia to 13 mo										
No	1971 (95.5)	17.2	1		24.1	1		17.4	1	
Yes	94 (4.4)	29.8	**2.05 (1.30-3.24)**	**.002**	63.8	**5.57 (3.61-8.60)**	**<.001**	11.7	0.63 (0.33-1.20)	.158
Child geohelminths§										
No	1452 (71.1)	17.5	1		24.5	1		17.7	1	
Yes	590 (28.9)	18.6	1.08 (0.84-1.38)	.538	30.0	**1.32 (1.07-1.64)**	**.010**	16.2	0.90 (0.69-1.16)	.422

ORs and 95% CIs were estimated using logistic regression. *P* values of less than .05 are shown in boldface. Ethnicity of “other” represents 1518 Mestizo/6 indigenous. Numbers of missing values are for maternal allergy (n = 13) and child geohelminth infections (n = 27).

*SPT*, Allergen skin prick test reactivity to any of 10 allergens.

∗ Socioeconomic status *(SES)* represents tertiles of *z* scores obtained using a factor analysis, with 1 representing the lowest and 3 representing the highest SES.

† Household overcrowding is defined as the number of persons living in the household per sleeping room.

‡ Any of cows, pigs, mules, donkeys, and horses.

§ Geohelminth infections in the child: presence of any geohelminth infection detected during the first 2 years of life.

**Table II:** Adjusted analyses for associations between maternal geohelminth infections or potential confounders and study outcomes

Variable	Eczema to 3 y		Wheeze to 3 y		Allergen SPT reactivity at 3 y	
Adjusted OR (95% CI)	*P* value	Adjusted OR (95% CI)	*P* value	Adjusted OR (95% CI)	*P* value
Any maternal geohelminth						
No	1		1		1	
Yes	1.26 (0.98-1.61)	.067	1.02 (0.82-1.27)	.837	0.79 (0.61-1.01)	.060
Maternal ethnicity						
Non–Afro-Ecuadorian	1		1		1	
Afro-Ecuadorian	1.25 (0.96-1.62)	.103	**1.30 (1.03-1.65)**	**.026**	1.12 (0.85-1.47)	.413
Maternal educational level						
Illiterate	1		1		1	
Complete primary	0.83 (0.58-1.16)	.275	0.87 (0.64-1.18)	.368	0.80 (0.56-1.14)	.225
Complete secondary	0.98 (0.64-1.49)	.915	0.89 (0.61-1.30)	.540	0.67 (0.43-1.04)	.073
Area of residence						
Urban	1		1		1	
Rural	1.07 (0.81-1.43)	.627	0.91 (0.71-1.18)	.477	1.00 (0.75-1.34)	.987
Sex						
Male	1		1		1	
Female	0.90 (0.71-1.14)	.373	**0.77 (0.63-0.95)**	**.014**	0.91 (0.72-1.15)	.439
Socioeconomic status∗						
1	1		1		1	
2	0.86 (0.64-1.15)	.301	0.84 (0.65-1.09)	.188	1.03 (0.77-1.39)	.827
3	0.85 (0.62-1.17)	.313	1.04 (0.79-1.37)	.785	1.05 (0.76-1.46)	.750
Birth order						
First	1		1		1	
Second-fourth	1.09 (0.81-1.46)	.573	1.26 (0.97-1.64)	.080	0.87 (0.64-1.14)	.287
≥Fifth	0.85 (0.57-1.28)	.441	1.18 (0.83-1.67)	.357	0.74 (0.50-1.10)	.137
Maternal allergy						
No	1		1		1	
Yes	**2.29 (1.46-3.61)**	**<.001**	1.34 (0.85-2.12)	.208	1.33 (0.80-2.23)	.276
Household overcrowding†						
No	1		1		1	
Yes	1.25 (0.96-1.61)	.094	1.15 (0.91-1.44)	.239	1.08 (0.83-1.14)	.564
Pets inside house						
No	1		1		1	
Yes	**1.32 (1.02-1.71)**	**.036**	1.15 (0.92-1.46)	.221	0.98 (0.75-1.29)	.909
Contact with large farm animals‡						
No	1		1		1	
Yes	0.94 (0.72-1.22)	.653	0.88 (0.70-1.11)	.291	0.79 (0.60-1.04)	.089
Pneumonia to 13 mo						
No	1		1		1	
Yes	**2.05 (1.27-3.29)**	**.003**	**5.64 (3.61-8.81)**	**<.001**	0.59 (0.30-1.15)	.122
Geohelminth infections in child§						
No	1		1		1	
Yes	0.98 (0.75-1.28)	.869	1.22 (0.97-1.54)	.096	0.92 (0.70-1.21)	.542

ORs and 95% CIs were estimated using logistic regression and adjusted for all variables shown. *P* values of less than .05 are shown in boldface. *SPT*, Allergen skin prick test reactivity to any of 10 allergens.

∗ Socioeconomic status *(SES)* represents tertiles of *z* scores obtained by using a factor analysis, with 1 representing the lowest and 3 representing the highest SES.

† Household overcrowding is defined as the number of persons living in the household per sleeping room.

‡ Any of cows, pigs, mules, donkeys, and horses.

§ Geohelminth infections in the child: presence of any geohelminth infection detected during the first 2 years of life.

**Table III:** Adjusted analyses for associations between types of maternal geohelminth infections and study outcomes, including SPT reactivity to HDMs

Geohelminth variable	Eczema to 3 y			Wheeze to 3 y			Allergen SPT reactivity at 3 y			SPT reactivity to HDM at 3 y		
Percent	OR (95% CI)	*P* value	Percent	OR (95% CI)	*P* value	Percent	OR (95% CI)	*P* value	Percent	OR (95% CI)	*P* value
Maternal *A lumbricoides* presence												
No (72.5%)	16.2	1		24.1	1		18.3	1		10.0	1	
Yes (27.5%)	21.8	**1.36 (1.04-1.77)**	**.026**	30.6	1.27 (1.00-1.61)	.052	14.2	**0.70 (0.52-0.95)**	**.021**	5.3	**0.48 (0.31-0.74)**	**.001**
Intensity												
Uninfected (77.8%)	16.6	1		24.2	1		18.0	1		9.8	1	
Light (17.1%)	21.7	1.32 (0.97-1.81)	.075	29.9	1.27 (0.96-1.68)	.092	13.1	**0.65 (0.45-0.94)**	**.022**	5.2	**0.47 (0.27-0.81)**	**.007**
Moderate/heavy (5.1%)	21.0	1.23 (0.71-2.10)	.459	34.3	1.54 (0.97-2.47)	.069	16.2	0.97 (0.54-1.73)	.918	4.8	0.53 (0.20-1.40)	.103
Maternal *T trichiura* presence												
No (71.1%)	16.9	1		25.1	1		17.8	1		9.6	1	
Yes (28.9%)	17.7	1.11 (0.84-1.45)	.463	27.9	1.02 (0.80-1.29)	.885	15.6	0.89 (0.67-1.18)	.537	6.4	0.77 (0.53-1.13)	.189
Intensity												
Uninfected (73.8%)	17.0	1		25.5	1		17.8	1		9.7	1	
Light (22.0%)	18.9	1.04 (0.77-1.39)	.811	25.9	0.87 (0.67-1.14)	.331	15.3	0.86 (0.63-1.17)	.324	6.7	0.72 (0.47-1.11)	.133
Moderate/heavy (4.2%)	24.7	1.48 (0.84-2.60)	.167	28.2	0.93 (0.54-1.61)	.806	12.9	0.81 (0.41-1.61)	.554	2.4	0.28 (0.07-1.19)	.086
Maternal hookworm presence												
No (94.4%)	17.4	1		26.1	1		17.3	1		8.8	1	
Yes (5.6%)	24.4	1.35 (0.85-2.16)	.205	23.5	0.71 (0.44-1.14)	.153	14.2	0.89 (0.51-1.56)	.690	7.1	1.07 (0.50-2.29)	.864

ORs and 95% CIs were estimated using logistic regression. *P* values of less than .05 are shown in boldface. ORs are controlled for maternal ethnicity and educational level, area of residence, sex, socioeconomic status, birth order, maternal allergy, household overcrowding, pets inside the house, contact with large farm animals, pneumonia to 13 months, geohelminths in the child, and the presence of other types of maternal geohelminth parasites. Uninfected data for prevalence are derived from the results of all parasitologic methods, whereas those for infection intensity were derived from Kato-Katz results.
